# Absence of canonical mutations in pediatric essential thrombocytosis: a case series

**DOI:** 10.1007/s44313-024-00036-4

**Published:** 2024-10-17

**Authors:** Jae Wook Lee, Suejung Jo, Jae Won Yoo, Seongkoo Kim, Nack-Gyun Chung, Bin Cho

**Affiliations:** 1 Division of Pediatric Hematology and Oncology, Department of Pediatrics, College of Medicine, The Catholic University of Korea, Seoul, Republic of Korea; 2 Department of Pediatrics, Seoul St. Mary’s Hospital, College of Medicine, The Catholic University of Korea, Seocho-Gu, Banpo-daero 222, Seoul, 06591 Republic of Korea

## Abstract

Essential thrombocytosis (ET) is a rare myeloproliferative disease in children, and there are few standard management guidelines. We herein report a case series of 10 pediatric patients with ET diagnosed at our institution over a period of 13 years. All patients fulfilled the World Health Organization diagnostic criteria for ET, and none harbored the canonical ET mutations *JAK2* V617F, *CALR*, or *MPL*. Overall, 7 of the 10 patients received treatment for ET, and during follow-up, 3 of these 7 patients discontinued cytoreductive therapy. No patient experienced hemorrhagic or thrombotic complications. Our case series emphasizes that the genetic features of pediatric ET may differ significantly from those of adult ET, and that treatment cessation is a possibility for some patients.

## Letter to the Editor

Essential thrombocytosis (ET) is a rare myeloproliferative disease (MPD) in children, with a reported annual incidence of 0.6 per 100,000 patients per year in the pediatric population [1]. The incidence of ET in South Korea is similarly low, with 2.53 cases per million reported in a cohort of patients aged 1–39 years [2]. Overall, key findings from previous studies on pediatric ET indicate a lower incidence of clonal mutations in *JAK2* V617F, *CALR*, and *MPL* than that in adults, with mutations in these genes identified in 23.6%–66.7% of patients [3–6]. Pediatric patients who lack canonical mutations may not have clonal MPD requiring lengthy treatment, and studies have reported the spontaneous resolution of thrombocytosis during follow-up [3, 7]. These unique characteristics of pediatric ET indicate that the utilization of treatment guidelines that mostly target adult ET populations may be inadequate for children. However, a consensus on optimal management is lacking, and further research is necessary to provide evidence-based guidelines for pediatric ET. Herein, we report the clinical and genetic characteristics of 10 patients with ET who were diagnosed at our institution over a period of more than 10 years.

Patients diagnosed with ET at the Department of Pediatrics, Seoul Saint Mary’s Hospital, from April 2009 to October 2022 were included in this retrospective study (*N* = 10; males, 6). This study was approved by our institutional ethics committee. Patient follow-up was conducted until December 31st, 2023.

Regarding clinical history, one of the patients had a family history of thrombocytosis. All patients underwent bone marrow examination and fulfilled the 2016 World Health Organization diagnostic criteria for ET [8]. The mean age at diagnosis was 12.3 years (range: 6.4–18.5 years), and the mean platelet count at diagnosis was 1,480 × 10^9^/L (range: 850–2,693 × 10^9^/L). Only three patients showed symptoms at presentation: headache and jaw pain (*n* = 1), headache and vomiting (*n* = 1), and headache and dizziness (*n* = 1). Screening for genetic mutations was performed either through real-time polymerase chain reaction and Sanger sequencing (*n* = 5) or through a targeted next-generation sequencing (NGS) panel comprising more than 80 genes related to myeloproliferative/myelodysplastic diseases (*n* = 5) using previously reported methods [9, 10]. All patients were evaluated for *JAK2* V617F mutation and were negative. None of the eight and seven patients who respectively underwent *MPL* and *CALR* genetic evaluations had a mutation in these genes. A targeted NGS study undertaken in five patients showed likely pathogenic mutations in *JAK2* exon 20 p.Arg867Gln (*n* = 1) and *ABCA12* (*n* = 1).

Five patients started either aspirin or cytoreductive therapy at diagnosis, and overall seven received some form of therapy during follow-up (Table 1). The mean platelet count at the start of treatment was 1,700 × 10^9^/L (range: 1,067–2,645 × 10^9^/L). The mean initial platelet count was significantly higher in patients who received treatment than in those who did not (1,720 vs. 923 × 10^9^/L, *P* = 0.033).

**Table 1:** Overall patient treatment

	*N* = 10 (%)
No. of patients who started treatment at diagnosis	5 (50)
No. of patients who received treatment overall	7 (70)
Mean platelet count at start of treatment (× 10^9^/L) (range)	1,700 (1,067–2,645)
Medications used	
Aspirin, hydroxyurea, anagrelide	2 (20)
Hydroxyurea	2 (20)
Aspirin, hydroxyurea, anagrelide, vincristine	1 (10)
Aspirin, hydroxyurea	1 (10)
Aspirin	1 (10)

Notably, four patients stopped therapy during follow-up: three who stopped cytoreductive therapy (Table 2) and one who only received aspirin for 2 weeks. All three patients stopped cytoreductive therapy at a higher than normal platelet count and did not reinitiate cytoreductive therapy (Table 2). None of the patients experienced hemorrhagic or thrombotic complications during follow-up.

**Table 2:** Patients who stopped cytoreductive treatment

	Medications	Duration of cytoreductive treatment (months)	Platelet count at treatment cessation (× 10^9^/L)	Platelet count at last follow-up (× 10^9^/L)
UPN 4^a^	Anagrelide (12.4 months) --> Anagrelide/hydroxyurea (8.7 months) --> Hydroxyurea (107.8 months)	129.0	887	726
UPN 6^a^	Hydroxyurea (13.0 months)	13.0	693	364
UPN 8	Hydroxyurea (67.7 months)	67.7	901	1,001

^a^Patients also received intermittent aspirin

The incidence of diagnostic genetic mutations is high in adult patients with ET, with approximately 90% having mutually exclusive *JAK2*V617F, *CALR,* or *MPL* mutations [11]. The incidence of these mutations is much lower in the reported pediatric ET cohorts, underscoring the key differences between adult and pediatric patients with ET. In our series of 10 patients, none had the canonical *JAK2* V617F mutation, and although the evaluation was incomplete, none had mutations in either *CALR* or *MPL*. One patient who underwent targeted NGS was diagnosed with *JAK2* exon 20 p.Arg867Gln, which has previously been reported in the context of pediatric Philadelphia chromosome-like acute lymphoblastic leukemia [12] but which has not been reported in ET. Comprehensive NGS analysis of mutations related to MPD may provide insights into the role of genetic mutations other than those in the three canonical genes in pediatric ET. Adult patients having ET with *JAK2* V617F mutation have a greater likelihood of developing thrombosis [13], and the lack of key ET mutations in our case series may have contributed to the absence of hemorrhagic or thrombotic complications.

Consensus guidelines recommend treatment for adult patients with ET if they have features consistent with a high risk of thrombosis, such as age > 60 years, cardiovascular risk factors, a history of thrombosis, or *JAK2* V617F mutation [14]. Pediatric patients rarely fulfill the adult-based criteria for high-risk ET, and the optimal treatment for pediatric ET remains unclear. Previous studies have reported spontaneous resolution of thrombocytosis in four pediatric patients with ET during follow-up for 17 years in the longest instance [3, 7]. Hence, a strategy of observation without treatment may be sufficient, although continued extreme thrombocytosis may contribute to an increased incidence of vascular complications such as thrombosis or hemorrhage. We emphasize that in our cohort of patients, cytoreductive therapy was stopped in three out of seven patients who received treatment overall at 13, 67.7, and 129 months, respectively, after the start of therapy. Unlike patients having ET with a genetic basis who may require prolonged treatment without cessation, pediatric patients with ET who lack canonical ET mutations may show resolution of thrombocytosis during follow-up.

We draw attention to the limitation of our study, namely that only seven of ten patients were fully evaluated for the three canonical ET mutations. Analysis of a larger number of patients may further clarify the incidence of pathogenic mutations in pediatric ET, and this preliminary study may be used as the basis for a nationwide study on pediatric ET. Although our NGS panel was comprehensive in the coverage of MPD-related genes, it did not include mutations such as *THPO* which have been implicated in familial thrombocytosis [15]; future evaluation of these mutations may be relevant in patients with a family history of thrombocytosis.

In summary, genetic evaluation of 10 pediatric patients with ET diagnosed over a period of 13 years at our institution showed that none of the patients had canonical ET genetic mutations. Cessation of cytoreductive treatment was possible in three of the seven patients treated overall. These diagnostic and treatment-related features underscore the differences between pediatric and adult ET and may influence the overall treatment strategy in such patients.

## Data Availability

No datasets were generated or analysed during the current study.
